# *TRIM67* Promotes the Proliferation, Migration, and Invasion of Non-Small-Cell Lung Cancer by Positively Regulating the Notch Pathway

**DOI:** 10.7150/jca.38286

**Published:** 2020-01-01

**Authors:** Jun Jiang, Hongjiu Ren, Yitong Xu, Muli Wudu, Qiongzi Wang, Zongang Liu, Hongbo Su, Xizi Jiang, Yao Zhang, Bo Zhang, Xueshan Qiu

**Affiliations:** 1 Department of Pathology, First Affiliated Hospital and College of Basic Medical Sciences, China Medical University, Shenyang, China.; 2 Department of Thoracic Surgery, Shengjing Hospital, China Medical University, No. 36 Sanhao St., Heping District, Shenyang, China.

**Keywords:** non-small-cell lung cancer, *TRIM67*, cell proliferation, cell migration, cell invasion, Notch pathway

## Abstract

Tripartite motif-containing 67 (TRIM67), an E3 ubiquitin ligase, belongs to the TRIM protein family. The relationship between TRIM67 and tumorigenesis is not fully clear. Here, we elucidated TRIM67 function in non-small cell lung cancer (NSCLC). TRIM67 immunostaining results were correlated with clinicopathological features. Moreover, the function of TRIM67 in cultured NSCLC cells was evaluated by MTT, colony formation, and Transwell assays. TRIM67 expression was associated with tumor size, lymph node metastasis, p-TNM stage, cancer cell differentiation, and poor prognosis. We altered TRIM67 expression in A549 and H1299 cell lines, and the results showed that TRIM67 promoted cell proliferation, migration, invasion and EMT by positively regulating the Notch pathway. Collectively, the results showed that TRIM67 promotes NSCLC progression through the Notch pathway and that TRIM67 expression is associated with clinicopathological features, indicating that TRIM67 may play an important role in promoting the development of NSCLC and could be applied as not only an important prognostic biomarker but also a therapeutic target in NSCLC.

## Introduction

Lung cancer is one of the leading causes of death worldwide, is closely associated with lung disease, and its incidence continues to rise 1-5. The 5-year survival rate remains at 15% and the prognosis of non-small cell lung cancer (NSCLC) patients is mainly related to tumor metastasis, which is related to transcriptional regulation of some key genes 5, 6-8. Therefore, understanding the mechanism of NSCLC progression is very important for the treatment of NSCLC.

The Notch pathway is involved in development and cell proliferation, differentiation, and apoptosis. As a result of its multiple effects on tissue homeostasis and cancer, this signaling pathway has attracted increasing attention as a potential therapeutic target. Notch proteins belong to a highly conserved family of cell surface receptors. Ligand binding leads to the complex proteolysis of these receptors by the γ-secretase complex, followed by translocation of the active intracellular incision nuclei and transcriptional activation domains 9. Notch signaling is activated by a ligand that binds to a receptor, initiating an intercellular communication system of five ligands (Delta-like 1, Delta-like 3, Delta-like 4, Jagged-1, and Jagged-2) and four receptors (Notch1-4). Ligand binding causes conformational changes of Notch, resulting in the exposure of the S2 site and sequence cleavage by the protease, disintegrase, a member of the metalloproteinase family and gamma secretase complex, to release the Notch intracellular domain (NICD). Finally, NICD is transferred to the nucleus, where it induces the target gene 10, 11 Yitong et al. 2018).

Tripartite motif-containing (TRIM) proteins are characterized by a common domain consisting of a RING finger, one or two B-box motifs and a coiled-coil motif, and are involved in many biological processes, including innate immunity, viral infection, cancer, and development 12-14. TRIM67 belongs to the tripartite motif (TRIM) protein family 15. Therefore, TRIM67 may be a specific E3 ubiquitin ligase, which is significant for target protein ubiquitin-dependent degradation or stabilization 16. TRIM67 was reported to be associated with the nervous system 17-19, but its function in tumors has not been reported.

In this study, we demonstrated that TRIM67 promotes the proliferation, migration, invasion and EMT of NSCLC cells through the Notch pathway. Additionally, we detected the expression of TRIM67 in NSCLC tissues and cell lines by immunohistochemistry and western blotting and altered the expression of TRIM67 in NSCLC cells to study the variations of the cancer-related phenotype and determine its role in NSCLC.

## Methods

### Specimens and patient data

These assays were performed as described previously 5. All procedures performed in studies involving human participants were in accordance with the ethical standards of the institutional and with the 1964 Helsinki declaration and its later amendments or comparable ethical standards, and informed consent was obtained from all individual participants.

### Immunohistochemistry

These assays were performed as described previously 5.

### Cell culture

These assays were performed as described previously 5.

### Plasmid construction, transfection, and administration of Notch pathway inhibitors

According to the manufacturer's instructions, Lipofectamine 3000 reagent was largely used for cell transfection (Invitrogen, Carlsbad, CA, USA). We used short hairpin RNA (shRNA) targeting *TRIM67* to knockdown the expression of *TRIM67*, which was cloned into the SGU6 vector (Sangon Biotech, Shanghai), for 48 h. For upregulating the expression of *TRIM67*, we used *TRIM67*-expression plasmid and the corresponding empty pCMVNeo04 vector (LongqianBiotech, Shanghai, China).

To inhibit Notch signaling, we treated cells with 4 µM DAPT (MedChemExpress, Monmouth Junction, NJ, USA), a γ-secretase inhibitor blocking the Notch pathway, in dimethyl sulfoxide (DMSO), and an equal amount of DMSO was accordingly supplied to cells as a control 24 h after transfection.

### Immunocytochemistry

Lung cancer cells cultured in 24-well plates for 24 h were immobilized in paraformaldehyde (4%) for 15 min and permeated with 0.1% Triton X-100 for 10 min. Next, the cells were washed with PBS and then blocked in bovine serum albumin (5%) for 1 h and incubated with anti-TRIM67 antibody (1:50) at 4 °C overnight. Thereafter, the cells were incubated with the secondary antibody, TRITC binding antibody, for 2 h; cell nuclei were re-stained with DAPI, and images were captured using the Olympus FV1000 laser scanning confocal microscope (Olympus, Tokyo, Japan).

### Western blotting

Western blotting revealed several functional proteins that directly affect cell migration, cell invasion 28-30, and cell proliferation 31, 32. Cellular proteins were extracted using lysis buffer (P0013; Beyotime Biosciences, Shanghai, China) containing protease-inhibitor cocktail (B14002; Biotool, Shanghai, China). Proteins were quantified using the Bradford method. Approximately 60 µg of each protein was separated by sodium dodecyl sulfate polyacrylamide gel electrophoresis on 10% gels, transferred to polyvinylidene fluoride membranes (Millipore, Billerica, MA, USA), blocked in 5% skim milk (232100; Becton Dickenson, Franklin Lakes, NJ, USA) at 28 ºC, and incubated with antibodies (Table 1) overnight at 4 ºC. Membranes were then washed and incubated with horseradish peroxidase-conjugated anti-mouse/rabbit IgG (1:2000; ZSGB-BIO, Beijing, China) at 37 ºC for 2 h. Finally, immunoreactivity was detected using the BioImaging system (UVP, Inc., Upland, CA, USA).

### Cell migration and invasion analyses

These assays were performed as described previously 5.

### Cell proliferation and colony formation assays

These assays were performed as described previously 33.

### Statistical analysis

Statistical analyses were mostly performed using SPSS 17.0 (SPSS, Inc., Chicago, IL, USA). Survival rates were analyzed by the Kaplan-Meier method and compared by the log rank test (Kaplan-Meier Plotter. Available from URL: http://www.kmplot.com/analysis/index.php?p=service&cancer=lung) 34^.^ Student's t-test was used to analyze statistically significant differences between the expression of TRIM67 and clinical features, and P < 0.05 was considered statistically significant.

## Results

### *TRIM67* correlates with clinicopathological characteristics and poor prognosis

**Figure 1:**
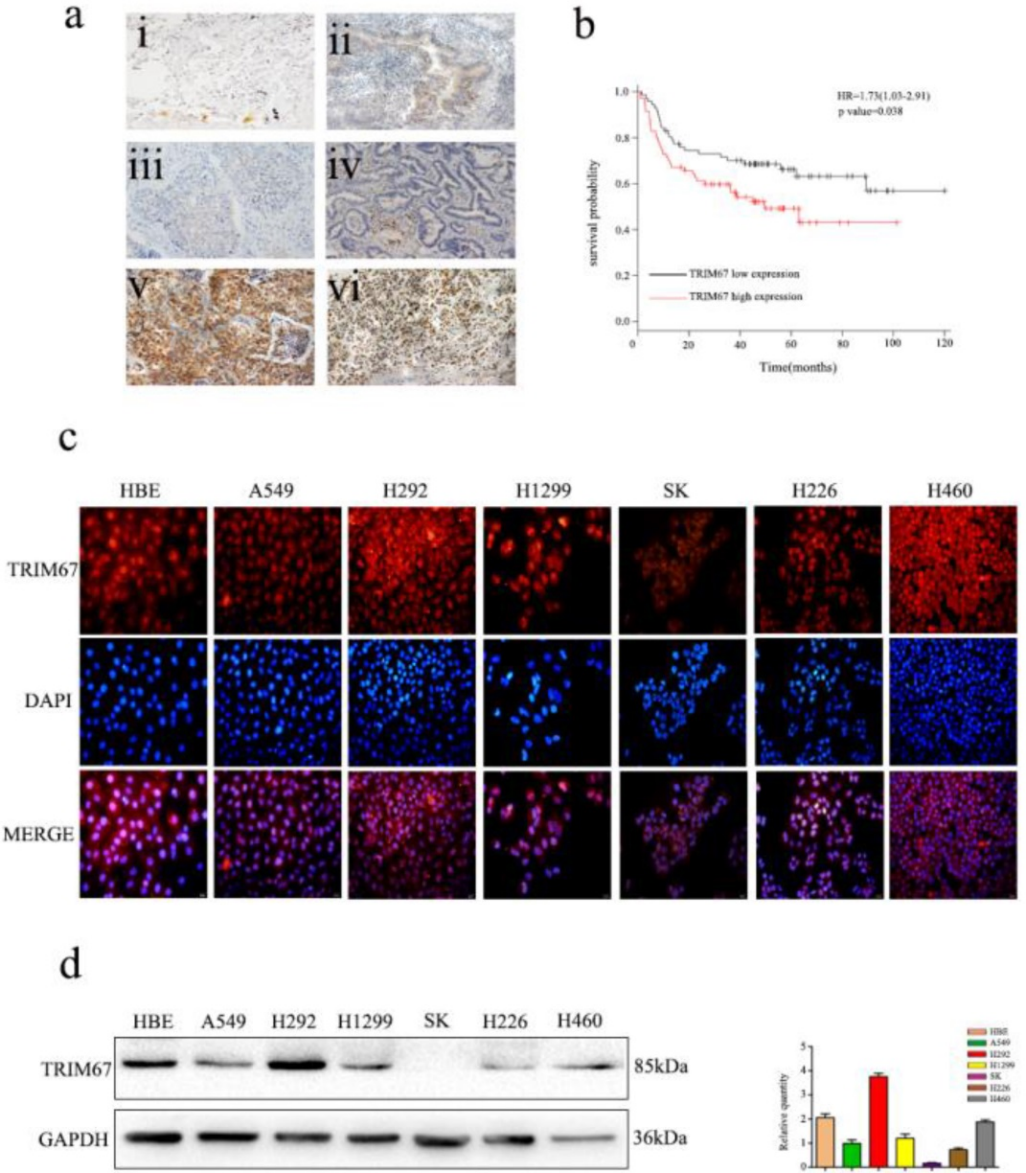
Expression of TRIM67 in non-small cell lung cancer (NSCLC) is associated with poor clinical prognosis. **a** TRIM67 protein levels were analyzed by immunohistochemistry in (i) alveolar cells, (ii) normal bronchial epithelial cells, (iii) well-differentiated squamous cell carcinoma, (iv) well-differentiated adenocarcinoma, (v) poorly differentiated squamous cell carcinoma, (vi) poorly differentiated adenocarcinoma. **b** Survival of NSCLC patients with high and low TRIM67 expression based on Kaplan-Meier curves. Hazard ratio (HR) and *P* value are indicated. **c** Immunofluorescence assays were performed to detect TRIM67 localization in human normal bronchial epithelial and NSCLC cell lines. TRIM67 was localized to the cytoplasm and nucleus. **d** TRIM67 levels in HBE cells and six NSCLC cell lines according to western blot analysis. Relative quantification analysis was based on gray scale values.

Immunofluorescence analysis of seven NSCLC cells showed that TRIM67 was localized in both the cytoplasm and nucleus (Fig. 1c). Moreover, immunohistochemical analysis showed that TRIM67 expression in low-differentiated NSCLC tissues was significantly higher than that in high-differentiated NSCLC tissues (Fig. 1a). Next, we investigated whether TRIM67 protein levels were associated with clinical features. The results showed that TRIM67 levels were positively correlated with differentiation (P < 0.001), tumor size (P = 0.018), lymph node metastasis (P < 0.001), and pathological TNM stage (P < 0.001), but not age (P = 0.424), sex (P = 0.328), and histological type (P = 0.514) as shown in Table 2. Moreover, high expression of TRIM67 was associated with lower survival in NSCLC patients according to the Kaplan-Meier database (Fig. 1b). Based on the above results, we suggest that TRIM67 might have an important effect on the biological functions of NSCLC.

Western blot analysis was also performed to determine the expression of TRIM67 in HBE normal bronchial epithelial cells and six NSCLC cell lines (Fig. 1d), and then we selected A549 and H1299 cell lines for subsequent studies because TRIM67 was moderately expressed in these two cell lines.

### TRIM67 expression promotes migration and invasion of NSCLC cells

**Figure 2:**
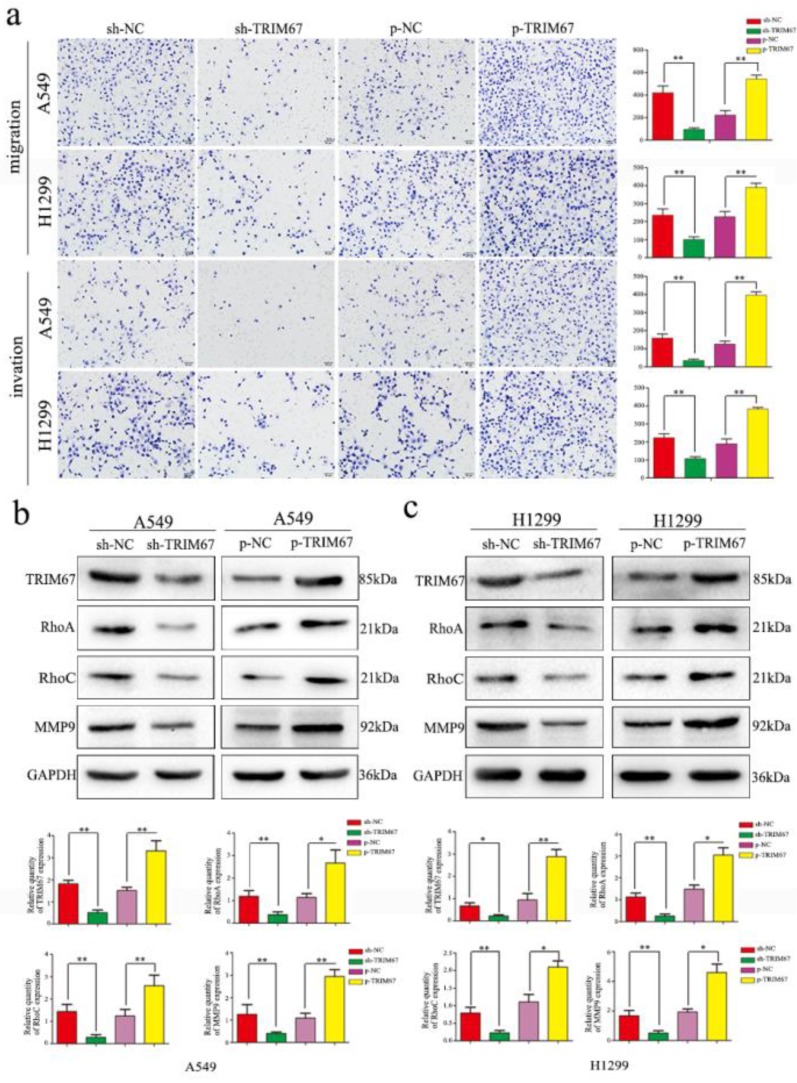
TRIM67 protein levels are positively correlated with non-small cell lung cancer (NSCLC) migration and invasion. **a** Transwell assays were performed to assess cell migration and invasion after TRIM67 overexpression and downregulation. TRIM67 overexpression in A549 and H1299 cells enhanced cell migration and invasion, whereas TRIM67 knockdown inhibited these processes. *P < 0.05; **P < 0.01.** b, c** Effects of TRIM67 levels on the expression of proteins associated with cell migration and invasion in A549 and H1299 cells. Relative quantification analysis was based on gray scale values. *P < 0.05; **P < 0.01.

Based on the previously mentioned findings, we next studied the effect of TRIM67 protein in NSCLC. To this end, we altered TRIM67 expression by downregulation or upregulation in both A549 and H1299 cell lines. We found that TRIM67 knockdown inhibited the migration and invasion of A549 and H1299 cells as compared to that of control cells through Transwell assays, whereas migration and invasion were promoted by TRIM67 overexpression (P < 0.05; Fig. 2a). Further, alteration of TRIM67 protein expression had an effect on the expression of proteins related to cell migration and invasion, consistent with the aforementioned results. Transfection efficiency was also examined by western blotting 48 h post-transfection. Upon TRIM67 upregulation, the levels of RhoA, RhoC, and matrix metalloproteinase (MMP)-9 increased, whereas its downregulation exerted the opposite effect (Fig. 2b and c). Consistent with our immunohistochemical results, these results suggested that TRIM67 enhances the migration and invasion of NSCLC cells.

### TRIM67 expression promotes the proliferation of NSCLC cells

**Figure 3:**
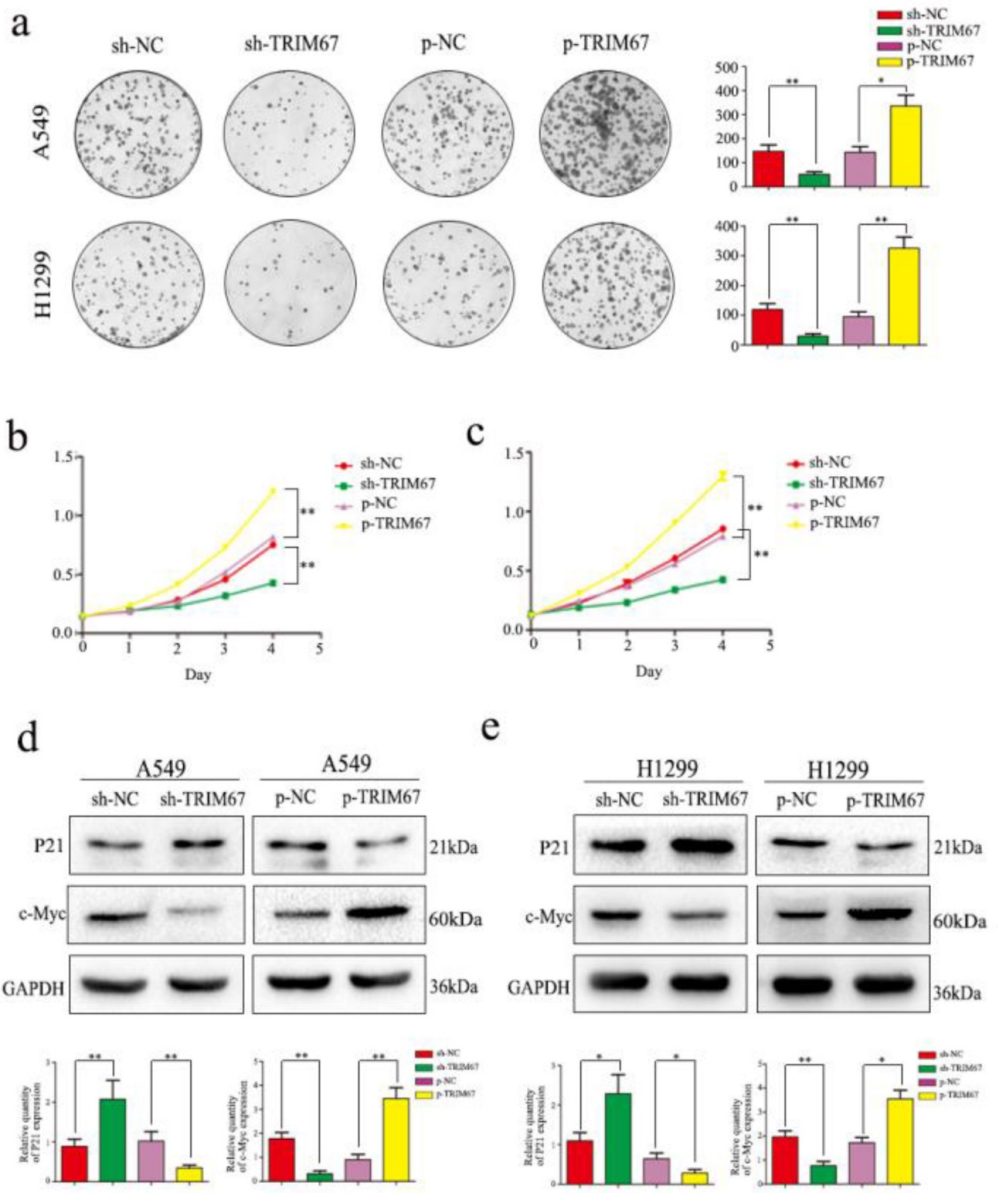
TRIM67 protein levels are positively correlated with non-small cell lung cancer (NSCLC) cell proliferation. **a** Colony formation assays were performed to assess cell proliferation after TRIM67 overexpression and downregulation. TRIM67 overexpression in A549 and H1299 cells enhanced cell proliferation, whereas TRIM67 knockdown inhibited cell proliferation. *P < 0.05; **P < 0.01. **b, c** MTT assays were performed to confirm that TRIM67 overexpression enhances cell proliferation and that TRIM67 knockdown inhibits this process. *P < 0.05; **P < 0.01. **d, e** The effects of TRIM67 upregulation or downregulation on the expression of proteins associated with cell proliferation in A549 and H1299 cells. Relative quantification analysis was based on gray scale values. *P < 0.05; **P < 0.01.

We next analyzed the effect of TRIM67 expression on cell proliferation. Colony formation analysis clearly demonstrated that TRIM67 expression and colony-formation ability of tumor cells were positively correlated in A549 and H1299 cells; specifically, downregulation inhibited clone formation, whereas upregulation had the opposite effect, when compared to that in respective control groups (Fig. 3a). Subsequent MTT assays revealed that upregulation of TRIM67 expression enhanced cell proliferation, whereas downregulation had an opposite effect in A549 cells (Fig. 3b), similar to the observation in H1299 cells (Fig. 3c). Consistent with these observations, TRIM67 overexpression promoted the proliferation of NSCLC cells, which also agreed with our immunohistochemical results showing that TRIM67 levels are associated with tumor size. We next analyzed the proliferation-related proteins, and the results showed that when TRIM67 was upregulated, the expression of c-Myc was also upregulated, whereas that of P21 was downregulated; knockdown of TRIM67 had the opposite effect on these proteins in both A549 and H1299 cells (Fig. 3d and e).

### TRIM67 positively regulates the expression of proteins related to epithelial-mesenchymal transition (EMT)

**Figure 4:**
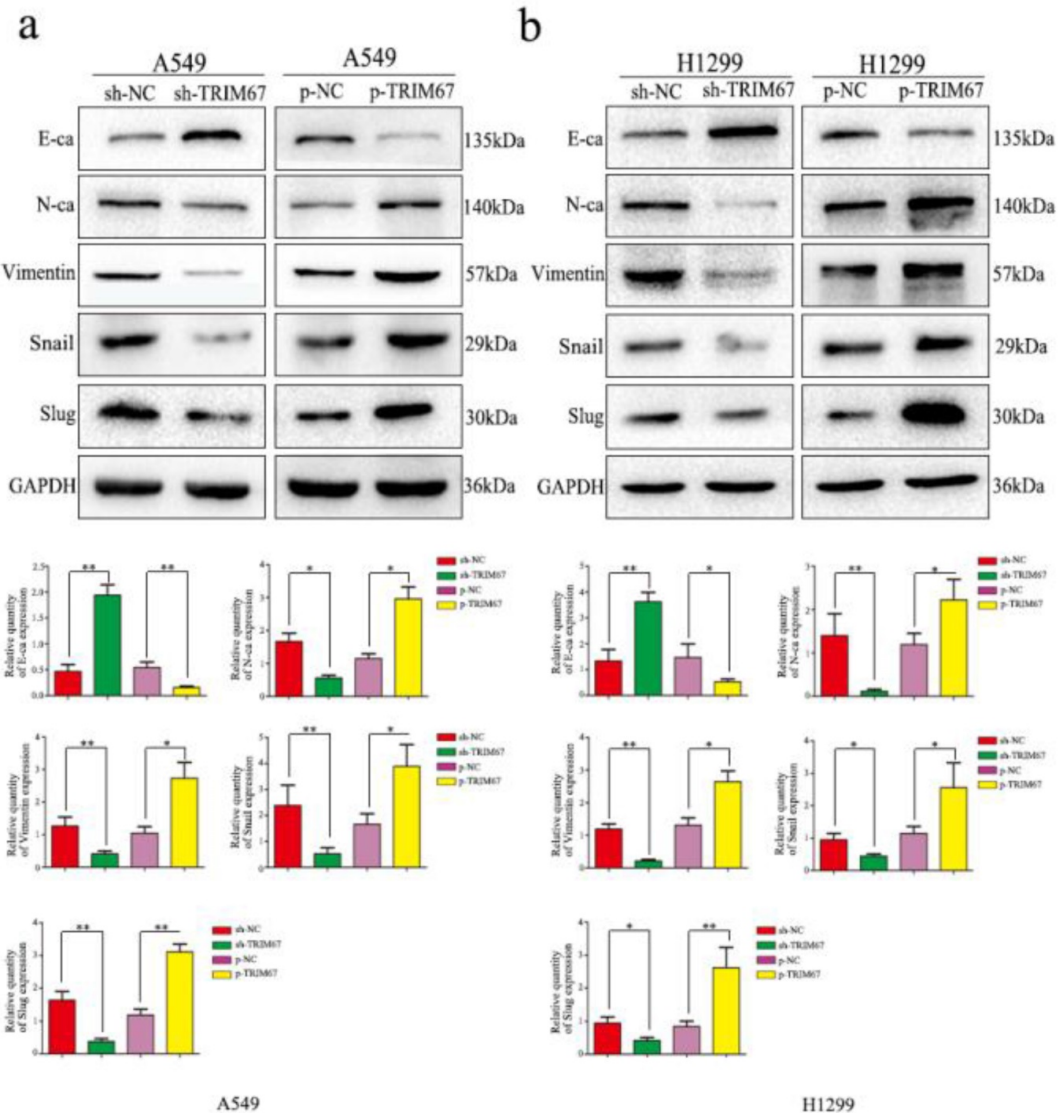
Effects of TRIM67 levels on the expression of epithelial-mesenchymal transition (EMT)-related proteins. **a** A549 and** b** H1299 cells were transfected with expression or shRNA vectors. Relative quantification analysis was based on gray scale values. *P < 0.05; **P < 0.01.

To elucidate the mechanism by which TRIM67 enhances the migration/invasion of lung cancer cells, we detected the expression of EMT-related proteins by western blotting. Results showed that increased TRIM67 expression enhanced N-cadherin, vimentin, Snail, and slug expression, but suppressed E-cadherin in A549 cells (Fig. 4a), similar to the observation in H1299 cells (Fig. 4b). These results revealed that TRIM67 can affect the migration and invasion of NSCLC cells by promoting epithelial-mesenchymal transition(EMT).

### TRIM67 regulates migration, invasion, cell proliferation, and EMT of NSCLC cells through the Notch pathway

**Figure 5:**
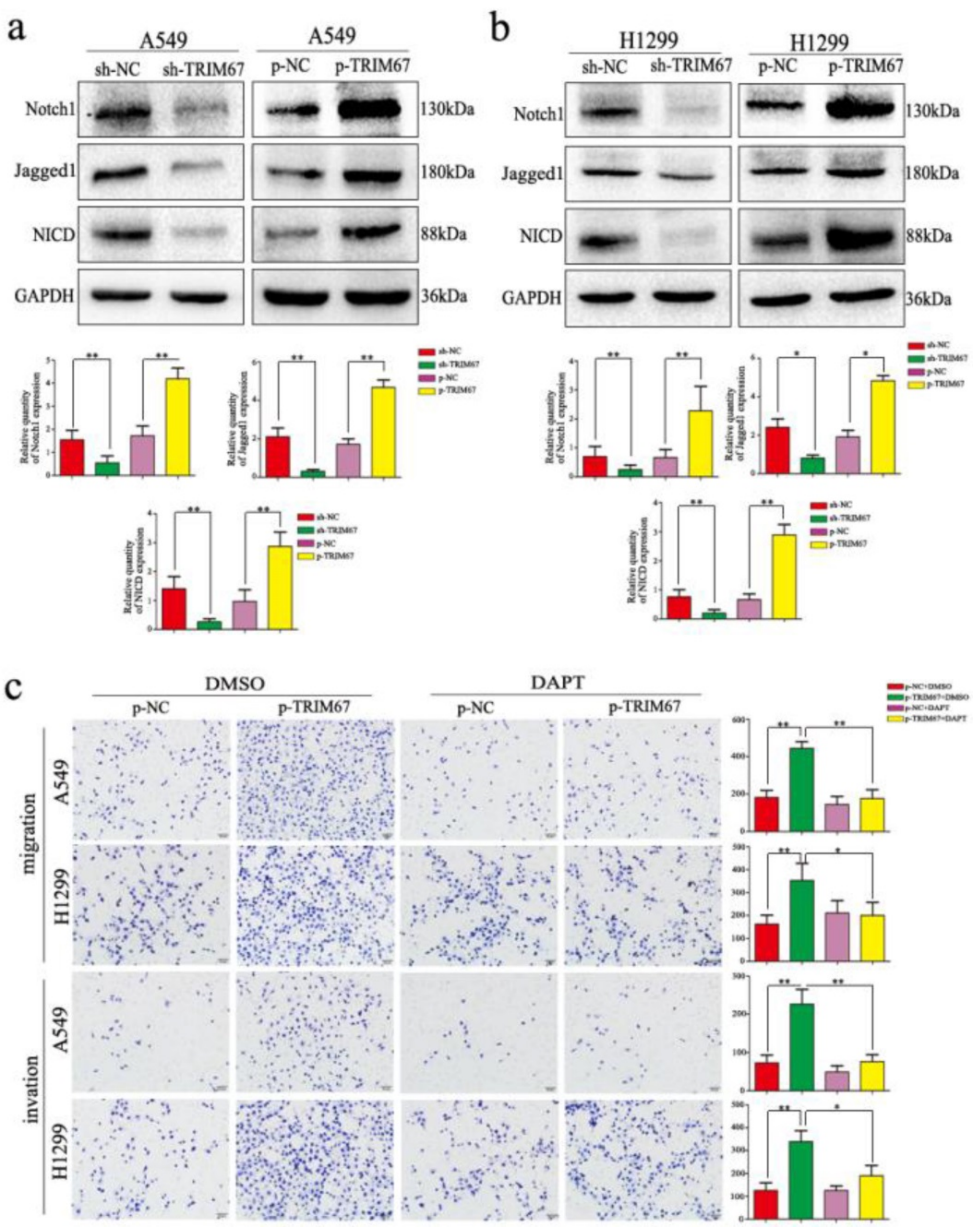
TRIM67 modulates Notch signaling to regulate cell migration and invasion in non-small cell lung cancer. **a**, **b** Changes in the expression of Notch-related proteins in **a** A549 and **b** H1299 cells. Relative quantification analysis was based on gray scale values. *P < 0.05; **P < 0.01. **c** Changes in the migration and invasion capacity of A549 and H1299 cells in the presence or absence of treatment with the Notch inhibitor DAPT and transfection with p-TRIM67. *P < 0.05; **P < 0.01.

**Figure 6:**
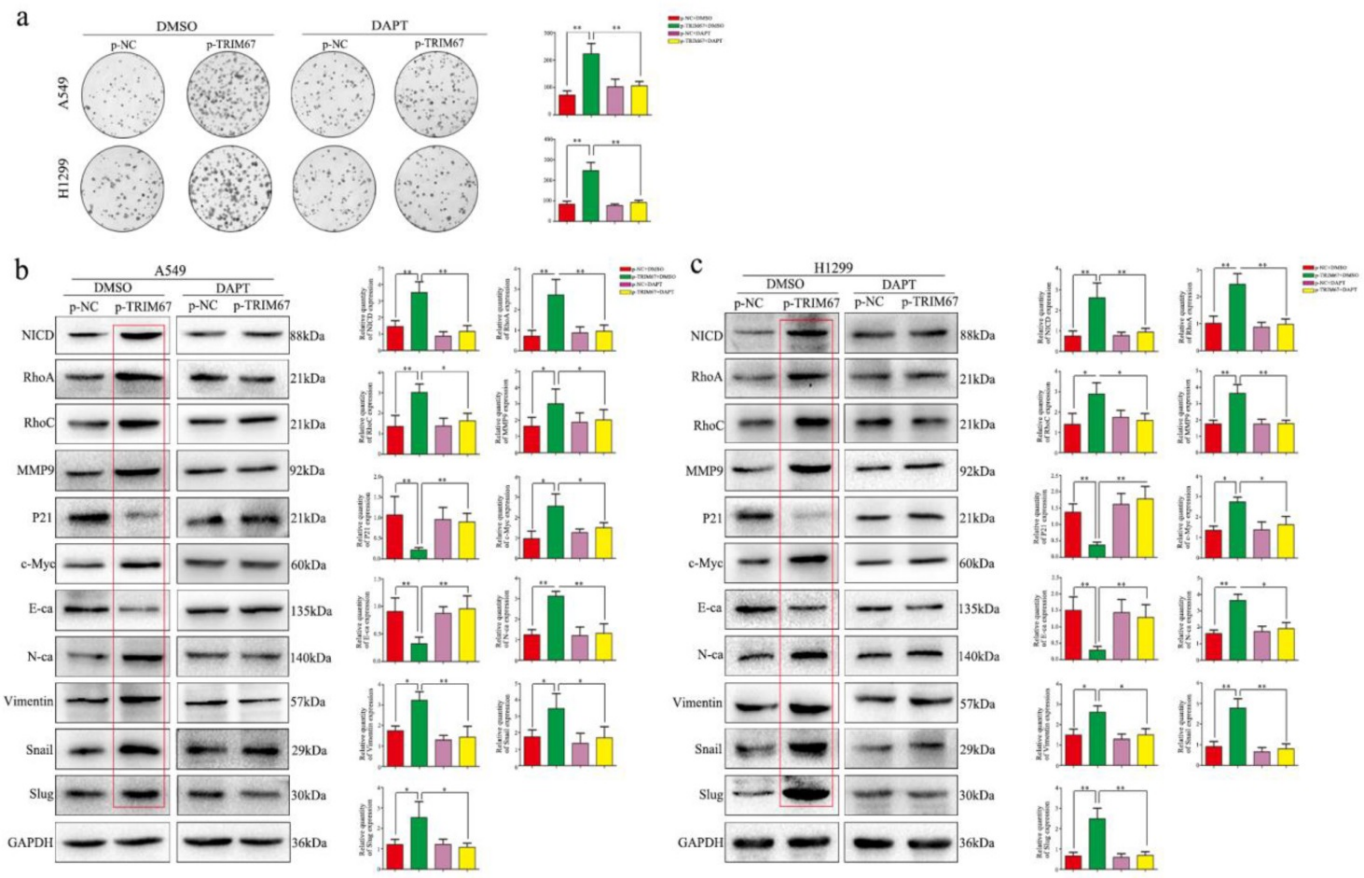
Notch signaling mediates the effects of TRIM67 on proliferation and epithelial-mesenchymal transition (EMT). **a** Changes in the proliferation activity of A549 and H1299 cells in the presence or absence of the Notch inhibitor DAPT after transfection with p-TRIM67. *P < 0.05; **P < 0.01. **b, c** Effect of TRIM67 in the presence or absence of Notch inhibition on protein expression in non-small cell lung cancer (NSCLC) cells. **b** A549 and **c** H1299 cells transfected with p-TRIM67 were treated with or without the Notch inhibitor DAPT and analyzed by western blotting for the expression of proteins involved in cell migration, invasion, proliferation, and EMT. Relative quantification analysis was based on gray scale values. *P< 0.05; **P< 0.01.

We clarified the role of TRIM67 in NSCLC cell migration, invasion, and proliferation and then investigated the biological mechanisms related to these effects. The Notch pathway is involved in the development of several cancers by influencing the expression of EMT-related proteins and other factors found to be altered by TRIM67 modulation; therefore, we examined whether TRIM67 is involved in the regulation of Notch signaling in NSCLC cells. Western blotting results showed that TRIM67 knockdown downregulated the expression of Notch1, Jagged1, and NICD in A549 and H1299 cell lines, whereas overexpression had the opposite effects (Fig. 5a and b). To investigate whether the function of TRIM67 was mediated through the Notch pathway, we used the inhibitor, DAPT, which inhibits NICD expression. DAPT inhibited cell migration, invasion, and proliferation in A549 and H1299 cells transfected with p-trim67 (Fig. 5c and 6a), and the effect of TRIM67 overexpression on protein expression of A549 and H1299 cells was reversed (Fig. 6b and c). These results indicated that activation of the Notch pathway contributes to TRIM67-mediated migration, invasion, and proliferation in NSCLC cells.

## Discussion

TRIM67 is a protein that belongs to TRIM family and is localized to the cytoplasm and nucleus. Here, we demonstrated that TRIM67 is expressed in NSCLC and multiple lung cancer cell lines.

In this study, we showed that the expression of TRIM67 was associated with a range of clinicopathological features, such as tumor size, differentiation, lymph node metastasis, p-TNM staging, and prognosis. We also demonstrated that TRIM67 promotes the proliferation, migration, invasion and EMT of NSCLC cells through the Notch pathway and the expression of downstream proteins such as RhoA, RhoC, MMP-9, P21, c-myc, and EMT-related markers 17, 20, 21, thereby influencing the malignant behavior of tumor cells. However, the mechanisms involved in these functions are unclear, and further studies are needed.

The role of the Notch pathway in cancers has been determined according to the effect of Notch pathway on cell proliferation, migration, and invasion 22-26. In the present study, we demonstrated that TRIM67 overexpression can upregulate Notch1, Jagged1, and NICD. NICD translocates to the nucleus, where it binds to several transcription factors and regulates the expression of target genes. The γ-secretase inhibitor DAPT blocks the Notch pathway by reducing the formation of NICD 27. When we added DAPT, the effects of TRIM67 on NSCLC functions were reversed, which confirmed that TRIM67 functions through the regulation of Notch signaling.

However, in this study, we did not confirm how TRIM67 acts on the Notch pathway. TRIM67 is an E3 ubiquitin ligase, so we speculate that this pathway is activated by ubiquitination of Notch pathway-inhibiting proteins. Even if this is true, it is still unclear which domain and binding site of TRIM67 are involved in this process, and thus further studies are needed.

In conclusion, our study demonstrated that TRIM67 plays a significant role in NSCLC via the Notch pathway, suggesting that TRIM67 might be a candidate prognostic biomarker and possible therapeutic target for NSCLC. Even though further studies are needed to determine the functions of TRIM67 in these biological processes, our results offer significant views and reflections about the functions and mechanisms of TRIM67 in tumorigenesis.

## Figures and Tables

**Table 1:** List of antibodies used for western blotting in the study.

Antibody name	Source	Catalog number	Host	Dilution
TRIM67 TRIM67	Sigma-Aldrich Sigma-Aldrich	HPA034776 SAB2103188	Rabbit Rabbit	1:50 1:100
GAPDH	Beyotime	AF0006	Mouse	1:1000
RhoA RhoC	Cell Signaling Technology Inc Cell Signaling Technology Inc	2117 3430	Rabbit Rabbit	1:500 1:500
MMP9	Cell Signaling Technology Inc	13667	Rabbit	1:500
P21	Cell Signaling Technology Inc	2947	Rabbit	1:1000
c-myc	Cell Signaling Technology Inc	13987	Rabbit	1:1000
E-Cadherin	Proteintech	20874	Rabbit	1:500
N-Cadherin	Proteintech	22018	Rabbit	1:500
Vimentin Snail Slug Jagged1 Notch1 NICD	Proteintech Cell Signaling Technology Inc Cell Signaling Technology Inc Cell Signaling Technology Inc Cell Signaling Technology Inc Wanlei Bio	10366 3879 9585 2620 3608 WL03097a	Rabbit Rabbit Rabbit Rabbit Rabbit Rabbit	1:500 1:500 1:500 1:500 1:500 1:500
HA	TransGen Biotech	HT301	Mouse	1:1000

**Table 2:** Association between TRIM67 expression and clinicopathological characteristics of non-small cell lung cancer patients.

Clinicopathological characteristics	Total N	*TRIM67*-positive	*TRIM67*-negative	*P* value
Age (years)				
≤ 60	148	81	67	
> 60	106	70	36	0.424
Sex				
Male	143	78	65	
Female	111	44	67	0.328
Histological type				
Squamous cell carcinoma	106	60	46	
Adenocarcinoma	148	88	60	0.514
Differentiation				
Well-moderate	127	44	83	
Poor	127	74	53	< 0.001
Tumor size (cm)				
≤ 5	224	98	126	
> 5	30	20	10	0.018
Lymph node metastasis				
Negative	150	52	98	
Positive	104	66	38	< 0.001
TNM stage				
I	148	50	98	
II-III	106	67	39	< 0.001

## Abbreviations

EMT: epithelial-mesenchymal transition
FBS: fetal bovine serum
IP: immunoprecipitation
NICD: notch intracellular domain
NSCLC: Non-small cell lung cancer
TRIM: tripartite motif
